# Evaluation of the Dimensional Accuracy of 3D-Printed Aligners: An In Vitro Study Using Reverse Engineering Analysis

**DOI:** 10.3390/jfb17010022

**Published:** 2025-12-30

**Authors:** Samuele Avolese, Fabrizio Sanna, Simone Parrini, Giada Chiarello, Danila Lava, Ambra Sedran, Andrea Deregibus, Nicola Scotti

**Affiliations:** 1 Department of Mechanical and Aerospace Engineering, Polytechnic University of Turin, 10129 Torino, Italy; 2 Specialization School in Orthodontics, Department of Surgical Sciences, Dental School of the University of Torino, 10126 Torino, Italy; 3 Department of Surgical Sciences, Dental School of the University of Torino, 10126 Torino, Italy

**Keywords:** 3D scanner, digital models, digital simulation, 3-dimensional printing, post-processing

## Abstract

Background: This study aimed to investigate the dimensional deformation that can occur during the fabrication of a 3D-printed aligner made with the TC-85 DAC resin (Graphy Inc., Seoul, Republic of Korea) and determine if the manual removal of the print supports before final aligner curing affects the dimensional accuracy. Methods: 10 subjects with permanent dentition were selected, and a set of aligners was digitally designed using the uDesign Direct Aligner beta software (Graphy Inc., Seoul, Republic of Korea). Each aligner was 3D-printed using TC-85 DAC resin (Graphy Inc., Seoul, Republic of Korea) twice: one copy was produced removing the print supports before final curing, whereas the other was cured with the supports still attached. The aligners were digitized and compared to the original design of the digitally designed aligner using RMS and Inter-second molar distance data to identify variations between 3D-produced aligners and their respective digital design. Results: the comparison between aligners produced in two different ways was statistically significant with a *p*-value < 0.0001 for both the records used. Conclusions: the manual removal of the print supports before final curing affects the dimensional accuracy of aligners made by direct 3D printing, permanently altering the aligner’s internal geometry, confirming that post-processing conditions significantly affect dimensional stability.

## 1. Introduction

The introduction of 3D printing has progressively revolutionized the dental practice and allows, in the orthodontic field, to provide personalized care through the creation of clear aligners. Recently, procedures allowing the direct 3D printing of aligners have become increasingly widespread in the dental market, eliminating the need for them to be thermoformed on a previously printed model [1,2,3].

Recent studies reported that directly 3D-printed aligners show better fit, higher efficacy, and superior reproducibility [4,5]. It has also been found that 3D-printed aligners generate biologically compatible orthodontic forces in vitro. Moreover, they may offer improved ability to deliver constant forces within the traditional range considered optimal for orthodontic movement [6]; in addition, it has been observed by in vitro studies that the factors released by these aligners during aging in water for 14 days do not exhibit cytotoxicity against human gingival fibroblasts and do not affect intracellular ROS levels [7]. However, the literature has also begun to demonstrate that the biocompatibility of directly printed aligners can depend on printing parameters and post-curing conditions [8], highlighting the need to carefully control manufacturing protocols for different material thicknesses.

Recently, a light-curing polymer has been introduced to the dental market: the Graphy TC-85 DAC resin (Direct Aligner Clear, Graphy Inc., Seoul, Republic of Korea), which is attracting attention for the production of directly printed clear aligners. This material is characterized by physical properties that allow better adaptation and fewer changes in strength loss over time [4,5]. Its most distinctive feature, which represents a novelty among direct-printing materials, is the shape-memory property, allowing the material to return to its original shape after deformation; this characteristic allows the aligner to alter the elastic modulus when immersed in hot water, and then recover its initial shape with body temperature [9]. This allows the force exerted to remain constant for a longer period of time, and in this way, a single aligner would be able to complete an entire treatment phase, speeding up therapy and reducing the amount of material used for orthodontic treatment [9,10,11,12].

On the one hand, 3D printing of this material also allows for design modification and local thickness adjustment to selectively manage anchorage and facilitate specific orthodontic movements; this allows maximum customization and optimization of the fitting for each patient, thanks to the possibility of managing the geometry and thicknesses of the aligner according to the needs of the treatment, thus maximizing the advantage offered by 3D printing.

On the other hand, direct 3D printing technology still presents several limitations, including the need for technical expertise and costly equipment, as well as the current scarcity of approved materials and dedicated software for direct aligner fabrication. Moreover, the literature provides limited clinical and technical data on these materials, and ongoing research is focused on improving their reliability and expanding their availability.

To achieve optimal accuracy and efficacy of this material, it is essential to closely monitor and comply with laboratory procedures to avoid any deformation or cytotoxicity [13]. In addition, in order for these types of printers to be able to print a complex object such as an aligner, it is necessary to include adequate printing supports in its design, which give stability while avoiding distortion and which must be removed later, before final curing [4].

As indicated by the manufacturer of the Tera Harz TC-85 DAC resin (Graphy Inc., Seoul, Republic of Korea), after 3D printing the aligners must be detached from the printing platform and inserted inside a centrifuge in order to remove the excess of uncured resin; this post-processing step is mandatory as it is not recommended to remove excess by other methods such as washing in isopropyl alcohol, which is useful for other types of resin [9], as this may compromise the properties of the TC-85 DAC resin (Graphy Inc., Seoul, Republic of Korea). A further fundamental step is the manual removal of the print supports before proceeding to the subsequent final polymerization of the aligner, after which the properties that characterize this resin will be acquired [4].

Given these technological and material constraints, optimizing each stage of the 3D printing workflow is essential to ensure accurate and clinically effective aligners. In particular, post-processing procedures play a critical role in determining the final dimensional stability and fit of the printed appliance [14].

The aim of the present experimental study was to evaluate whether the manipulation required to remove the printing supports, when performed before the final curing of the aligner, induces deformations that persist after polymerization. To this purpose, the shape stability of directly 3D-printed clear aligners was assessed by comparing the inner surface of their digital design with the corresponding physical models printed in TC-85 DAC resin (Graphy Inc., Seoul, Republic of Korea), following in detail the manufacturer’s recommended protocol.

To isolate the effect of manual handling, aligners produced following the manufacturer’s protocol were compared with those cured without removing the printing supports.

The null hypothesis stated that there is no difference in deviation, with respect to the digitally planned inner surface, between the two post-processing protocols for aligners printed with TC-85 DAC resin (Graphy Inc., Seoul, Republic of Korea).

As a secondary analysis, differences between upper and lower arch aligners were also evaluated.

## 2. Materials and Methods

### 2.1. Sample Collection and Preparation

For this study, 10 subjects with complete permanent dentition were selected, without the presence of all third molars in the arch.

Selected subjects were not undergoing orthodontic treatment and had no prosthetic restorations, fixed retainers, carious lesions, or dental fillings. In addition, inclusion criteria also include the absence of dental crowding and occlusal wear.

The sample size was determined a priori by reference to previous in vitro studies investigating the dimensional accuracy of directly 3D-printed orthodontic aligners. In particular, Koenig et al. [10] employed comparable sample sizes to detect differences in geometric accuracy between manufacturing protocols. Based on the methodological similarity and exploratory nature of the present study, a sample size of 10 specimens per group was considered appropriate to ensure statistical reliability while maintaining experimental feasibility.

An intraoral scan was taken for each subject using the iTero Element 2 intraoral scanner (© 2020 Align Technology, Inc., Tempe, AZ, USA), and both digital models of the upper and lower jaw were exported in STL format. A pair of clear aligners (one for the upper arch and one for the lower arch) was designed for each subject, without any type of movement and without any type of attachment on the tooth surfaces. The 3D aligners were designed using the uDesign Direct-Aligner beta software (Graphy Inc., Seoul, Republic of Korea), programming a thickness of 0.50 mm and an offset value of 0.05 mm for digital design [15,16]. The same software was used to automatically generate the printing support structures, keeping the default settings: the aligner was tilted by 40° relative to the build platform with the posterior portion closer and the anterior portion farther from the build plate, the “Intensity” value was set to 30, and the minimum distance between supports was 1.4 mm.

The aligners designed in this way, with their supports, were exported to STL files and placed on a single print file. The following direct printing was performed via a Sprint Ray Pro95 printer (SprintRay Inc., Los Angeles, CA, USA) at 100 μm thickness per layer, using Tera Harz TC-85 DAC resin (Graphy Inc., Seoul, Republic of Korea).

Each aligner type was printed twice, resulting in two pairs of upper and lower aligners. The printer was set to the “SprintRay US—SprintRay Splint” program.

When the print was complete, the aligners were detached from the print platform using the scraper given by the printer manufacturer. The excess resin was removed through 2 centrifugation cycles of 6 min each and a subsequent manual drying using cotton pellets.

Subsequently, the two copies of each aligner pair were processed by a single operator in two different ways:

1. For the first copy, the print supports were manually removed using finger pressure and then inserted into the curing machine (G1).
2. For the second copy, the print supports were not removed, but the aligners with all the supports were placed directly in the Tera Harz Cure curing machine (G2).

The aligners were then cured in a nitrogen-saturated environment [17,18] for 14 min using the Cure M machine (Graphy Inc., Seoul, Republic of Korea), as indicated by the manufacturer’s standard guidelines [19], and let cool naturally for at least 1 h [4]. After that, an aligner tester (printed more than the previous ones at each printing cycle only for this final check) was immersed in boiling water for 1 min, in order to verify that it did not become opaque (a sign that involves the invalidity of the processed aligners), as indicated by the manufacturer.

### 2.2. Reverse Engineering Workflow and Analysis

The outer surface of each designed aligner was digitally removed using Geomagic Control X software (version 2020.1.1, © 2020 3D Systems, Inc., Rock Hill, SC, USA), leaving only the inner surface of the virtual aligner in STL format.

**Figure 1:**
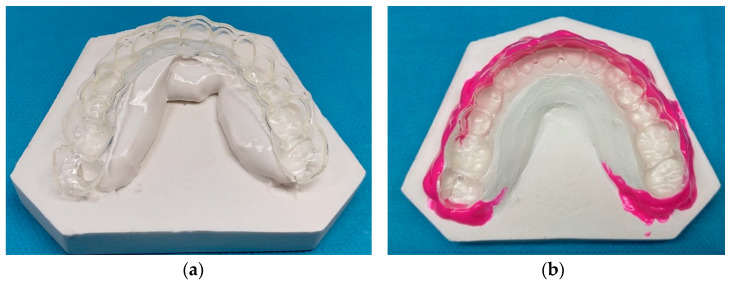
Aligner positioning on a rigid plaster support (**a**); removal of undercuts using light-type silicone (**b**).

From the physical copy of the 3D-printed aligners, an impression of the internal surface of each aligner was taken in this way: once the final polymerization was finished, the aligner was in fact passively positioned on special rigid plaster supports useful for stabilizing it during the casting of the plaster and the undercuts were removed using a light type silicone (Elite H-D+ Super-Fast Light Body—Zhermack SpA, Badia Polesine, Italy.) (Figure 1).

**Figure 2:**
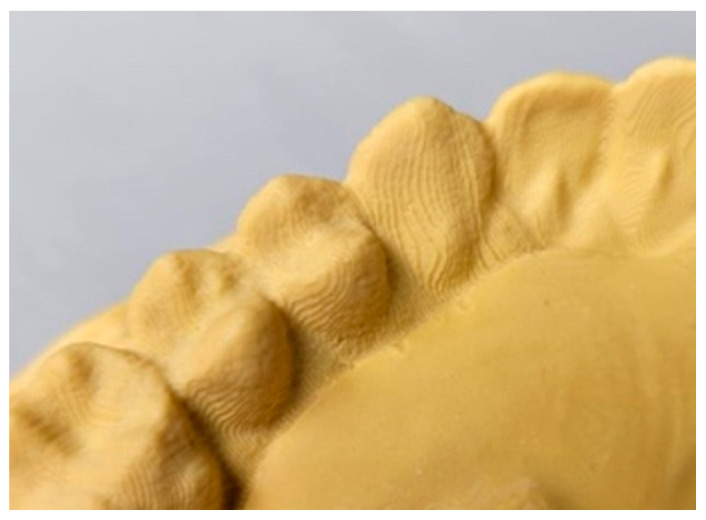
Dentations of the inner surface of the aligner. Note the ability to detect surface striations of the aligner due to the 3D printing process.

Then, using a vacuum mixer, the extra-hard plaster was poured onto the inner surface of the aligner (Figure 2), obtaining the plaster cast [20]. This cast was then digitized using the Lab scan inEosX5 scanner (Dentisply Sirona, Charlotte, NC, USA). Direct scanning of transparent aligners was not feasible due to optical reflection and low surface readability; therefore, plaster replicas were used to avoid applying opacifying agents that could artificially alter the internal aligner thickness. The use of extra-hard dental stone mixed under vacuum helped standardize the procedure and minimize possible errors during setting.

The STL file of the inner surface of the aligner obtained from digitizing the precision impression was compared with the corresponding inner surface of the digitally designed 3D aligner. G1 and G2 were compared using best-fitting matching of the Geomagic Control X software (version 2020.1.1, © 2020 3D Systems, Inc.).

For this purpose, the RMS (Root Mean Square) was used as a statistical indicator of the average magnitude of deviations between the two compared STL files [20,21]; in this context, RMS represents the mean surface deviation between the test model and the reference geometry, providing a quantitative measure of overall trueness.

As suggested by the study published by Nabil Muhsen Al-Zubair [22], the intermolar distance at the level of the second molars was calculated by a single expert operator using Geomagic Control X software (version 2020.1.1, © 2020 3D Systems, Inc.). The difference, in absolute value, between the intermolar distance measured on the STL files of the 3D-printed aligners and the same distance measured on the corresponding digital projects was thus calculated. This reference was chosen because distal regions of the aligner are more prone to dimensional changes, making second molars a sensitive indicator of shape accuracy. Moreover, the greater absolute distance reduces the relative impact of measurement variability compared with more anterior landmarks.

### 2.3. Statistical Analysis

The values were noted on an Excel spreadsheet (Version 16. 86 © 2024 Microsoft, Redmond, WA, USA) following the progressive order of the subjects.

SPSS software (Version 24.0, IBM Corporation, 1 New Orchard Road, Armonk, NY, USA) was used for statistical analysis. Quantitative data are reported as mean ± standard deviation (SD). Comparisons between the paired conditions (aligners cured with supports vs. supports removed) were performed using two-tailed paired *t*-tests. The Fisher exact test was applied to compare results between upper and lower arches. A significance level of *p* < 0.05 was adopted for all analyses.

## 3. Results

**Figure 3:**
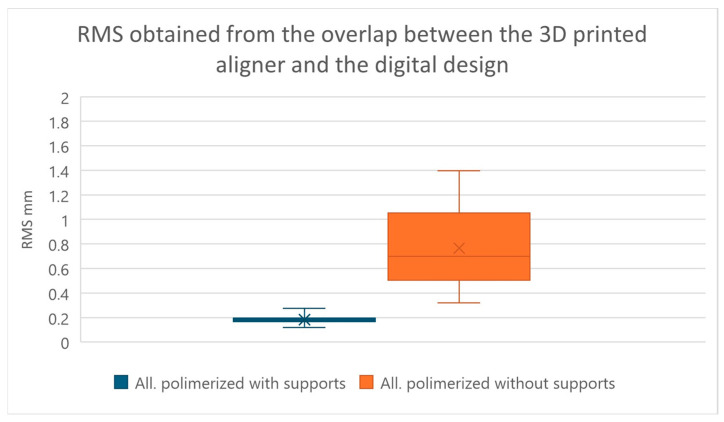
Graph showing RMS values obtained from comparing aligners that were cured without removing print supports and aligners that were cured after having removed the supports. In the graph, the box represents the interquartile range, the central line the median, the X the mean, and the whiskers the minimum and maximum values.

The results obtained from the comparison between 3D-printed aligners with their corresponding digital design, considering the RMS value [23,24], are summarized in Table 1. We find that the comparison between G1 and G2 was statistically significant with a *p*-value < 0.0001, using the two-tailed paired *t*-test (significance level of 5%) (Figure 3).

The null hypothesis that there is no difference in removing the print supports before or after final curing is therefore refuted, with an extremely significant result (*p*-value < 0.001).

**Figure 4:**
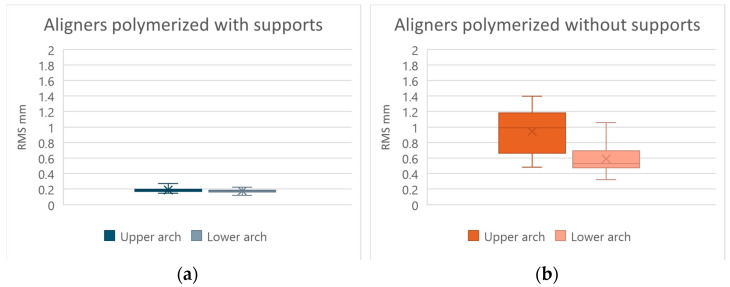
Graphs showing RMS obtained by comparing 3D-printed aligners with their respective digital designs between the values obtained from the upper arches and those obtained from the lower arches. Aligners cured with the print supports (**a**) and without the print supports (**b**). In the graph, the box represents the interquartile range, the central line the median, the X the mean, and the whiskers the minimum and maximum values.

Moreover, the comparison between the values obtained from the upper arches and those obtained from the lower arches, using the Fisher exact test (with a significance level of 5%), did not give statistically significant results both with regard to the protocol in which the aligners were cured with all supports and with regard to the one in which the supports were removed before the polymerization, having been obtained *p*-values of 0.4955 and 0.2346, respectively (Figure 4). Therefore, for both 3D-printed aligner production protocols, the null hypothesis that there are no differences between the results obtained in the upper arch and those obtained in the lower arch is confirmed, since there is not a sufficient level of significance (*p*-value > 0.05).

**Figure 5:**
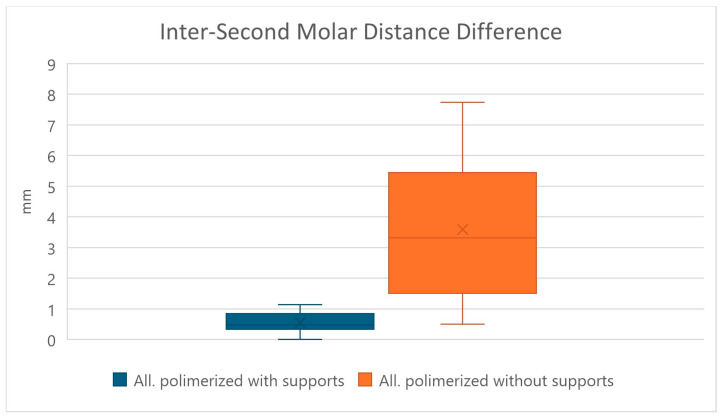
Difference of the inter-second molar distance obtained by comparing the aligners obtained by 3D printing with the corresponding digital project. In blue, the difference found measuring the aligners cured with the supports, and in orange, the difference found measuring the aligners cured without the printing supports. In the graph, the box represents the interquartile range, the central line the median, the X the mean, and the whiskers the minimum and maximum values.

The results obtained from the comparison between 3D-printed aligners with their corresponding digital design, considering the value of the intermolar distance at the level of the second molar of the 3D-printed aligner compared to the corresponding measurement of the digital design, are summarized in Table 2. We find that the comparison between aligners obtained by curing them without removing the print supports and those obtained by curing them by first removing the print supports was statistically significant with a *p*-value < 0.0001, using the two-tailed paired *t*-test (significance level of 5%) (Figure 5).

This refutes the null hypothesis that there is no difference in removing the print supports before or after final curing, with an extremely significant result (*p*-value < 0.001).

**Figure 6:**
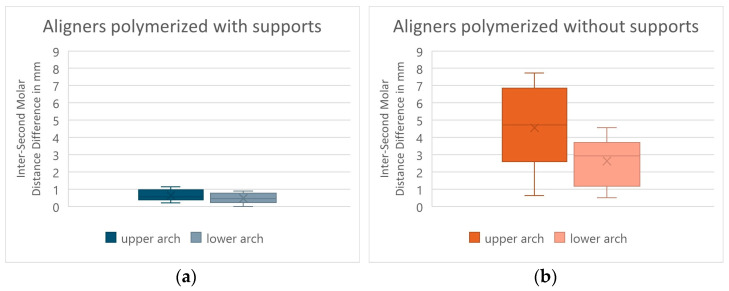
Inter-second molar distance difference value in mm obtained from the comparison of 3D-printed aligners with their respective digital designs between the values obtained from the upper arches and those obtained from the lower arches. Aligners cured with the print supports (**a**) and without the print supports (**b**). In the graph, the box represents the interquartile range, the central line the median, the X the mean, and the whiskers the minimum and maximum values.

The comparison (using the value from the inter-second molar distance obtained from the measurements made on the 3D-printed aligners and on the respective digital designs) between the values obtained from the upper arches and those obtained from the lower arches, using through the Fisher exact test (with a significance level of 5%), did not give statistically significant results both as regards the protocol in which the aligners were cured with all the supports and as regards the one in which all the supports were removed prior the final curing, giving us *p*-values of 0.9107 and 0.105, respectively (Figure 6). Therefore, for both 3D-printed aligner production protocols, the null hypothesis that there are no differences between the results obtained in the upper arch and those obtained in the lower arch is confirmed, since there is not a sufficient level of significance (*p*-value > 0.05).

## 4. Discussion

Recent evidence indicates that direct 3D printing of clear aligners and related orthodontic appliances can achieve clinically relevant levels of dimensional fidelity, but multiple factors in the digital workflow influence geometric accuracy. A recent systematic review reported that direct 3D printing of retainers and aligners generally achieves clinically acceptable trueness and precision, but accuracy outcomes are influenced by printer type, material, and processing variables [25]. Experimental work has also shown that specific fabrication parameters, such as print orientation, significantly affect the dimensional fidelity of printed aligners, with some orientations producing lower deviations from the digital design than others [26]. These findings emphasize the importance of evaluating specific manufacturing protocols when interpreting dimensional deviations in 3D-printed orthodontic appliances and provide context for the present comparison of post-processing strategies.

The aim of the present experimental study was to evaluate whether the alignment manipulation necessary to remove the supports, carried out before the final curing of the aligner, imparts deformations that persist even after the final aligner polymerization.

Statistical analysis revealed a significant difference between aligners cured with and without supports (*p* < 0.0001), indicating dimensional inconsistency between the two production protocols. This observation was found on all types of measurements made (RMS and difference in inter-second molar distance) with a *p*-value < 0.0001.

In general, aligners cured without supports showed greater variability than those cured with supports; this aspect also emerged by observing the values of the standard deviations, which are greater in the group including aligners in which the supports were removed. This indicates that in the group without supports, there is a greater dispersion of the results obtained, which deviate more from their average value, indicating a greater possibility of random error of the technique, precisely due to the manual removal of the print supports before final curing.

The statistical analysis allowed us to observe that the value of the RMS or “root mean square”, used in this study to evaluate the reliability and dimensional accuracy [23] is on average higher in aligners in which the supports were removed before polymerization, with a high standard deviation, showing a greater variability of the results in this group analyzed (mean 0.7656 mm ± 0.3106). The group, including the aligners in which the supports were kept during the final curing, showed a lower mean RMS value and a lower dispersion of the values, with the standard deviation being lower than the previous group (mean 0.1827 mm ± 0.0307).

**Figure 7:**
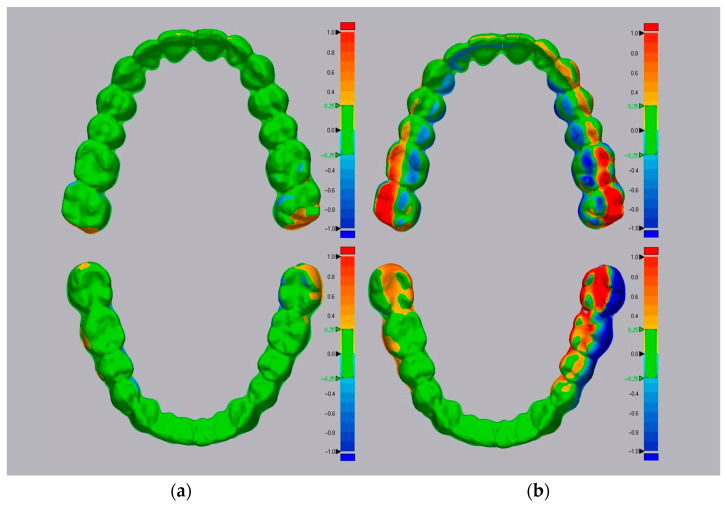
3D colorimetric maps obtained from the automatic superimposition of STL files using Geomagic software (version 2020.1.1, © 2020 3D Systems, Inc.), using a tolerance level of 250 μm; on the left (**a**) comparison between the aligners obtained by curing them without having removed the print supports with the original digital project, and on the right (**b**) the same comparison but using the aligners cured after the manual remotion of the printing supports.

Colorimetric deviation maps obtained through automatic STL superimposition in Geomagic software (version 2020.1.1, © 2020 3D Systems, Inc.) (Figure 7) visually confirmed these differences between printed aligners and their corresponding digital models. Based on these observations, the intermolar distance was evaluated at the level of the second molars, as the posterior region is most susceptible to transverse dimensional changes [22].

From the analysis of these quantitative measurements it can be seen that, as for the value previously analyzed, there is a substantial difference between the two protocols, namely, that the means of the intermolar distance at the level of the second molars are 0.5490 mm ± 0.3154 in the group of aligners in which the supports were kept during curing and 3.5895 mm ± 2.1902 in the group in which the supports were removed before curing.

Finally, no significant differences were detected between upper and lower arches (*p* > 0.05), suggesting that dimensional deviations are primarily influenced by the post-processing protocol rather than arch morphology.

Therefore, these findings suggest that removing printing supports before final curing increases the risk of aligner deformation.

A possible explanation is that manual support removal can induce slight mechanical stresses or distortions in the resin while it is still partially polymerized; these deformations may then be permanently fixed during the subsequent post-processing phase due to the viscoelastic behavior of the material.

From a clinical perspective, the observed deformations may compromise the precise fit of the aligner on the dental arches and lead to alterations in the internal geometry of the aligner; this may modify the distribution and magnitude of forces delivered to the teeth compared with the digitally planned force system, potentially leading to unintended or less predictable orthodontic movements.

A future idea to reduce deformation in the posterior area of the aligner could be to design a transversal support that connects the most posterior portions of the aligner to each other, that is rigid enough not to deform during the removal maneuvers of normal printing supports, and that needs to be removed only after the final curing of the 3D-printed aligner. The feasibility, printability, and potential dimensional effects of such solutions require dedicated investigation and were beyond the scope of the present study.

### Study Limitations

A potential limitation of this study could be the technique used for digital superimposition, which, being automatic, could lead to minimal non-calculable errors; for this reason, the measurement of the intermolar distance at the level of the second molars was used, but characterized by minimal intra-operator variability.

Another possible limitation of the study could be the use of a single thickness for 3D-printed aligners. Future studies could, therefore, analyze whether higher print thicknesses are better able to maintain stability during processing.

The aligners were designed without attachments and without programmed tooth movement, reflecting a passive aligner configuration rather than full clinical treatment conditions. This experimental setup was intentionally chosen to isolate the effect of post-processing protocols on intrinsic dimensional accuracy.

Moreover, direct mechanical testing of the material in a partially polymerized state was not conducted. Future studies should investigate the thermo-mechanical properties (e.g., elastic modulus, viscoelastic behavior, shape-memory recovery) of TC-85 DAC resin before and after final polymerization, using standardized methods as previously described in fully polymerized specimens [4], in order to quantify the in vitro observations that we have analyzed. Similarly, no thermal or mechanical aging procedures were performed; as a result, the present findings describe the initial dimensional accuracy of directly printed aligners after fabrication and post-processing, rather than their long-term shape stability under simulated intraoral conditions.

In addition, a starting point for future research could be to compare TC-85 DAC resin (Graphy Inc., Seoul, Republic of Korea) with other resins that do not require handling before final curing, as excesses are eliminated by washing in alcohol solution and supports are removed after final curing. Newer direct-print aligner materials, such as Graphy TA28 resin [8], may exhibit different polymerization kinetics and mechanical behavior, potentially leading to different dimensional outcomes.

As far as the development of the project is concerned, it will certainly be possible to increase the number of data involved to be able to obtain statistical data of greater relevance and more targeted analyses.

## 5. Conclusions

Within the limitations of this study, the findings indicate that the removal of printing supports before the final curing process leads to measurable deformations in 3D-printed clear aligners produced with TC-85 DAC resin (Graphy Inc., Seoul, Republic of Korea). These results demonstrate that manual manipulation before complete polymerization can permanently alter the aligner’s internal geometry, confirming that post-processing conditions significantly affect dimensional stability.

From a clinical perspective, even small deformations may compromise the fit and effectiveness of clear aligners, emphasizing the importance of adhering to optimized post-processing protocols that minimize manual handling before final polymerization.

Therefore, based on the present findings, workflows involving manual removal of print supports before final curing are not recommended.

Further studies will be needed to further investigate the topic and to evaluate the dimensional stability of 3D-printed aligners with resins.

## Figures and Tables

**Table 1:** RMS values obtained from comparing aligners that were cured without removing print supports and aligners that were cured after having removed the supports.

		RMS Value in mm	
		Aligners Polymerized with Supports	Aligners Polymerized Without Supports
Upper Aligners	specimen 1	0.167	0.5291
	specimen 2	0.1861	1.0361
	specimen 3	0.1451	0.4836
	specimen 4	0.1838	0.7799
	specimen 5	0.2743	0.7089
	specimen 6	0.1974	1.1263
	specimen 7	0.1958	1.1643
	specimen 8	0.2001	0.9462
	specimen 9	0.1707	1.3974
	specimen 10	0.1838	1.2367
Upper Aligners Mean		0.1904 mm ± 0.0338	0.9409 mm ± 0.3069
Lower Aligners	specimen 1	0.1773	0.7119
	specimen 2	0.1912	0.7119
	specimen 3	0.1818	0.4895
	specimen 4	0.1733	0.543
	specimen 5	0.1189	0.6857
	specimen 6	0.1774	0.5011
	specimen 7	0.1796	0.648
	specimen 8	0.2262	1.059
	specimen 9	0.1619	0.5144
	specimen 10	0.1623	0.4299
Lower Aligners Mean		0.1750 mm ± 0.0268	0.5904 mm ± 0.2031
Total Mean		0.1827 mm ± 0.0307	0.7656 mm ± 0.3106

**Table 2:** Difference of the inter-second molar distance obtained by comparing the aligners obtained by 3D printing with the corresponding digital project.

		Difference Inter-Second Molar Distance in mm	
		Aligners Polymerized with Supports	Aligners Polymerized Without Supports
Upper Aligners	specimen 1	0.38	0.63
	specimen 2	1.14	5.73
	specimen 3	0.2	1.23
	specimen 4	0.95	3.66
	specimen 5	0.62	3.05
	specimen 6	0.48	6.78
	specimen 7	0.41	7.08
	specimen 8	1.01	3.71
	specimen 9	0.37	7.73
	specimen 10	0.76	5.94
Upper Aligners Mean		0.6320 mm ± 0.3179	4.5540 mm ± 2.4709
Lower Aligners	specimen 1	0.25	0.85
	specimen 2	0.73	0.5
	specimen 3	0.88	2.21
	specimen 4	0.44	1.28
	specimen 5	0	3.16
	specimen 6	0.88	3.47
	specimen 7	0.47	2.9
	specimen 8	0.1	4.36
	specimen 9	0.33	4.56
	specimen 10	0.58	2.96
Lower Aligners Mean		0.4660 mm ± 0.3059	2.6250 mm ± 1.3980
Total Mean		0.5490 mm ± 0.3154	3.5895 mm ± 2.1902

## Data Availability

The full anonymized dataset of this study was opened and made available from the corresponding author when it is required.

## Abbreviations

The following abbreviations are used in this manuscript:

RMS	Root Mean Square
ROS	Reactive Oxygen Species
DAC	Direct Aligner Clear
